# Differences in Movement Pattern and Detectability between Males and Females Influence How Common Sampling Methods Estimate Sex Ratio

**DOI:** 10.1371/journal.pone.0159736

**Published:** 2016-07-21

**Authors:** João Fabrício Mota Rodrigues, Marco Túlio Pacheco Coelho

**Affiliations:** Programa de Pós-Graduação em Ecologia e Evolução da Universidade Federal de Goiás, Departamento de Ecologia, Universidade Federal de Goiás, Goiânia, GO, Brasil; Liverpool John Moores University, UNITED KINGDOM

## Abstract

Sampling the biodiversity is an essential step for conservation, and understanding the efficiency of sampling methods allows us to estimate the quality of our biodiversity data. Sex ratio is an important population characteristic, but until now, no study has evaluated how efficient are the sampling methods commonly used in biodiversity surveys in estimating the sex ratio of populations. We used a virtual ecologist approach to investigate whether active and passive capture methods are able to accurately sample a population’s sex ratio and whether differences in movement pattern and detectability between males and females produce biased estimates of sex-ratios when using these methods. Our simulation allowed the recognition of individuals, similar to mark-recapture studies. We found that differences in both movement patterns and detectability between males and females produce biased estimates of sex ratios. However, increasing the sampling effort or the number of sampling days improves the ability of passive or active capture methods to properly sample sex ratio. Thus, prior knowledge regarding movement patterns and detectability for species is important information to guide field studies aiming to understand sex ratio related patterns.

## Introduction

Nowadays, biodiversity is facing a major crisis caused by climate change and human modifications of natural habitats, which are pushing species towards extinction [1,2]. In such a scenario, a proper evaluation and sampling of biodiversity is mandatory.

There are currently many methods to sample terrestrial fauna, and they can be roughly classified into active methods, such as active searches (time-constrained searches, visual, and audio encounter surveys, for example), and passive methods, such as pitfall traps, glue traps, funnel traps, and mist nets [3–6]. Evaluating the efficiencies of these methods is an essential step in improving our ability to sample biodiversity. Many field studies have evaluated the efficiency of these methods regarding their ability to sample a region’s species composition and richness [6–8], and how these methods influence the estimates from species abundance distributions [3,4]. Other field studies have also evaluated how changes to the arrangement and characteristics of these methods, for example trap size and design, may influence their outcomes [9]. However, such evaluations are expensive, time consuming, and sometimes impossible to replicate.

On the other hand, simulation modelling allows biologists to explore the efficiency of sampling methods using a quasi-experimental approach. Using models to simulate ecological processes and evaluate the efficiency of sampling methods is known as a Virtual Ecologist (VE) approach [10]. A VE approach is one of the most efficient ways to test the performance of sampling methods, because it explores the efficiency of sampling methods against a known truth. By simulating a pattern that the researcher knows to exist, it is possible to evaluate if the sampling method of interest is capable of identifying the known pattern [11]. In natural environments, it is impossible to know the complete ‘*truth*’ because the observed patterns are based on limited data [11,12]. Thus, VE is an interesting approach to evaluate sampling methods of terrestrial vertebrates and has been recently used to test the efficiency of different arrangements of pitfall traps to sample biodiversity [13,14]. A similar approach can be used to evaluate if sampling methods are also efficient in reproducing important population data.

Sex ratio (the proportion of males and females in a given population, which are able to reproduce) is an important characteristic of natural populations, since strongly biased sex ratios may be problematic for population maintenance. Besides, knowledge of the sex ratio is also important to understand patterns of mating competition and sexual selection [15]. Although an equal sex ratio (1 male: 1 female) is most common in natural environments, some life history traits such as differences in mortality probability between adult and juvenile females and a longer maturation time in one of the genders might result in unequal sex ratios [16]. However, inadequate sampling methods may also result in the estimate of unequal sex ratios, even when the population has an equal proportion of males and females, and such problems are stronger when males and females have different probabilities of being captured using a particular sampling method [16,17]. Thus, assessing how efficiently the active and passive capture methods recover a population’s sex ratio is important knowledge for conservation and ecological studies.

This study aimed to evaluate the efficiency of passive and active capture methods in recovering sex ratios of populations under different scenarios of sampling effort, movement patterns, and detectability. To the best of our knowledge, this is the first study to use a computer simulation to evaluate the efficiency of different sampling methods to correctly reproduce populations’ sex ratios.

## Material and Methods

Our simulation was written using R programming language (R ver. 3.2.1), and the code is available as supplementary material (S1 Appendix). We simulated the movements of males and females from a single generation of a species in a gridded space in successive time steps, sampling individuals at each time step using procedures to simulate passive and active capture methods. Our simulation followed a mark-recapture approach, thus allowing us to know the identity of the sampled individuals.

### Model dynamics

First, individuals were randomly allocated in a grid of N x N, where N was the grid resolution. All simulations had an equal number of males and females. We assumed that the total number of individuals was n = a * N^2^. We parameterized a as being equal to four.

All individuals were allowed to move in each time step of our model. Males and females had movement probabilities which accounted for how easily they would move to another cell of the geographical domain. Male probability was m whilst for females, it was 1 –m. We defined female movement probability in this way in order to reduce the number of parameters in our model, and because it easily represented the scenarios we wanted to evaluate: m = 0.5 represents males and females with equal movement probabilities, m = 0.1 represent a large difference in movement pattern between males and females, and m = 0.3 is an intermediate difference. Once they moved, movements to a cell were distance mediated, and the probability of reaching a cell was equal to 1/d_ij_, where d_ij_ was the distance between the initial cell i and the possible new cell j. Distances between any given pair of grid cells (d_ij_) were calculated using Euclidian distance. Cells near the current cell of the individual will have a higher probability of receiving the individual. On the other hand, distant cells will have a low probability of receiving it. In other words, movement is dependent on the distance from the initial cell.

The edges of our simulated geographical domain were physical boundaries to individuals. We assumed the boundaries existed in our domain because empirical geographical domains are edge limited (e.g. forest fragments, islands, lakes, continents).

### Simulating Sampling Methods

In this study, we simulated active and passive capture methods, which are widely used to sample terrestrial vertebrates and invertebrates and are different in sampling efficiency. Active methods are usually biased by the observer’s sampling ability, habitat structure, and species detectability [3–5]. On the other hand, passive methods are not influenced by the observer’s experience and species detectability, but are restricted to species that could reach the traps (e.g. terrestrial traps will usually capture terrestrial organisms) [3,7]. In our simulation, we included the particular differences of each sampling method.

To simulate passive capture methods, we assumed that a single cell of the simulated geographical domain had a ‘perfect’ passive sampling method (a mist net or a pitfall trap station, for example). Thus, every individual that moves to a cell containing a sampling station was considered sampled. Sampling stations were distributed in space following a standard configuration. One or more transects were randomly selected in the geographical domain and sampling stations were located in individual cells of the transect, alternating so one cell had a trap and the next was without a trap (see supporting information and S1 Fig for a more detailed explanation regarding the passive sampling organisation used in this study). The number of randomly chosen transects depended on the sampling effort parameter that controlled the percentage of area in the domain that would receive the sampling method.

On the other hand, the active method consisted of a search performed by one or more observers for a species of interest or the entire assemblage. However, different species in an assemblage or the sexes within one species may have different detectability [16,17], which our simulations had to account for. In our simulation, one transect per sampling day was randomly chosen for active searches (see supporting information and S1 Fig for more explanations regarding this method, too). Although the number of pitfall lines could vary, we selected only one transect for active sampling because, while pitfall traps are fixed during the whole field survey, active search transects changed every sampling day, allowing this latter method to fully explore the studied area without needing to use more than one transect in a single day. Sampling occurred in every cell of the randomly selected transect. We simulated the active search method including variability in detectability of the sexes. Thus, active searches occurred in the randomly chosen transect and each individual present within the cell where the active search was set had a chance of being captured based on the detectability of each sex.

After all the sampling time steps, we evaluated if the estimated sex ratio based on captures of each method was different from 1:1 (population sex ratio) using a Chi-square test with P < 0.05. To evaluate the estimated sex ratio, each individual captured was considered only once, although it may have been captured many times during the simulation, which is appropriate as the model could recognize individuals.

### Parameter sensitivity

We initially established a set of default parameters and performed simulations allowing only one parameter to vary in order to explore the sensitivity of the model to each parameter. Prior to properly sampling the individuals in our gridded space using the different capture methods, we allowed the simulation to run 100 time steps to allow the individuals to cover the space and be distributed according to their movement characteristics, instead of being only randomly distributed. Each parameter combination was replicated 100 times, hereafter called “full simulation”. For each full simulation, the parameters varied in the following six ways: (i) Grid size ranged between a 10 x 10 cell grid up to a 55 x 55 cell grid, incrementing by five cells in both the rows and columns in each full simulation; (ii) Number of sampling days (time steps with sampling) varied from 35 to 350 days, increasing by 35 days after each full simulation; (iii) Movement probability of males (m) ranged from 0.5 (no difference between males and females—remember that movement probability of females is 1—m) to 0.95, increasing by 0.05 after each full simulation; (iv) Passive sampling effort was varied between 10% and 55% of the grid space, incrementing by 5% in each full simulation; (v) Detectability of males and females were kept equal and varied from 10% to 100%, increasing 10% in each full simulation; (vi) Detectability of males and females varied but keeping their sum equal to 1. For this, we started with male detectability equal to 10% and the female as 90%, changing by 10% in each full simulation.

To evaluate how sampling effort (number of sampling days and passive sampling effort) influenced the efficiency of the passive and active capture methods in scenarios with differences in movement pattern and detectability between males and females, we organised a second set of parameters. We reran the number of sampling days (ii from above) and passive sampling effort (iv from above) full simulations, but now under four different scenarios with differences in movement probability between the sexes and four different scenarios with differences in detectability between males and females.

All the values of parameters used and their results in both sets of simulations are available in the supporting information (S1 and S2 Tables).

## Results

**Fig 1:**
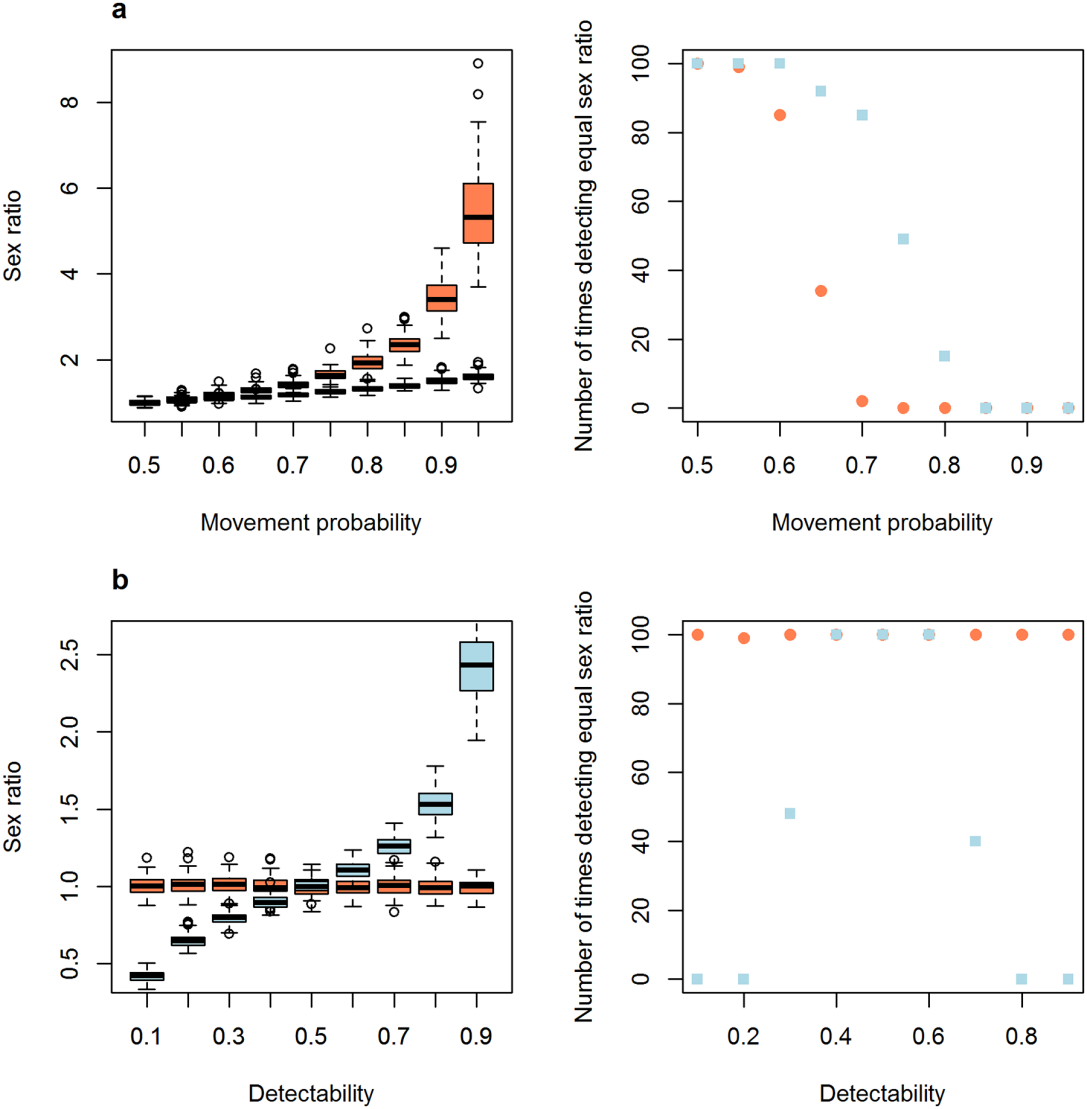
The influence of differences in movement pattern and detectability between males and females on sampling method efficiency in estimating a population’s sex ratio. Sex ratio distributions (left) and number of times sex ratio was recorded as equal (right) in 100 simulations when changing a) differences in movement pattern between males and females; b) differences in detectability between males and females. The number of times sex ratio was recorded as equal was determined by the number of times that the chi-square test performed on the sex ratio sampled in each simulation found a non-significant result. Red circles represent passive capture methods, and blue squares are active search.

In the first set of parameters used in our simulation to explore model sensitivity, grid size (affecting the number of individuals), number of sampling days, passive sampling effort and different levels of equal detectability in males and females did not affect the efficiency of passive and active searches to find an equal sex ratio (see Table 1 and Supporting Information for detailed results). When only these parameters varied, both sampling methods recovered an equal sex ratio in almost all the simulations. However, increasing the difference in movement pattern and detectability between males and females reduced the ability of the sampling methods to detect the population’s equal sex ratio, producing biases in the estimated sex ratios (Fig 1 and Table 1). Differences in movement patterns produced biased estimations of sex ratios for both sampling methods, but this effect was stronger for the passive method (Fig 1a). On the other hand, including differences in detectability between males and females produced biased sex ratio estimates only in active searches (Fig 1b), which is expected given the lack of influence of this parameter in our passive sampling simulation.

**Table 1:** Model sensitivity to each parameter. Parameters were individually modified in order to test their effect on the number of times sex ratio was correctly estimated. A negative effect means that an increase in the parameter caused a reduction in the number of times sex ratio was properly estimated. Movement probability and detectability were the only parameters that caused biased estimations of sex ratios.

Parameters	Effect on passive sampling	Effect on active sampling
Grid resolution (affecting the number of individuals)	No effect	No effect
Number of sampling days	No effect	No effect
Difference in movement probability	Negative effect	Negative effect
Passive Sampling effort	No effect	No effect
Equal detectability levels for males and females	No effect	No effect
Difference in detectability between males and females	No effect	Negative effect

**Fig 2:**
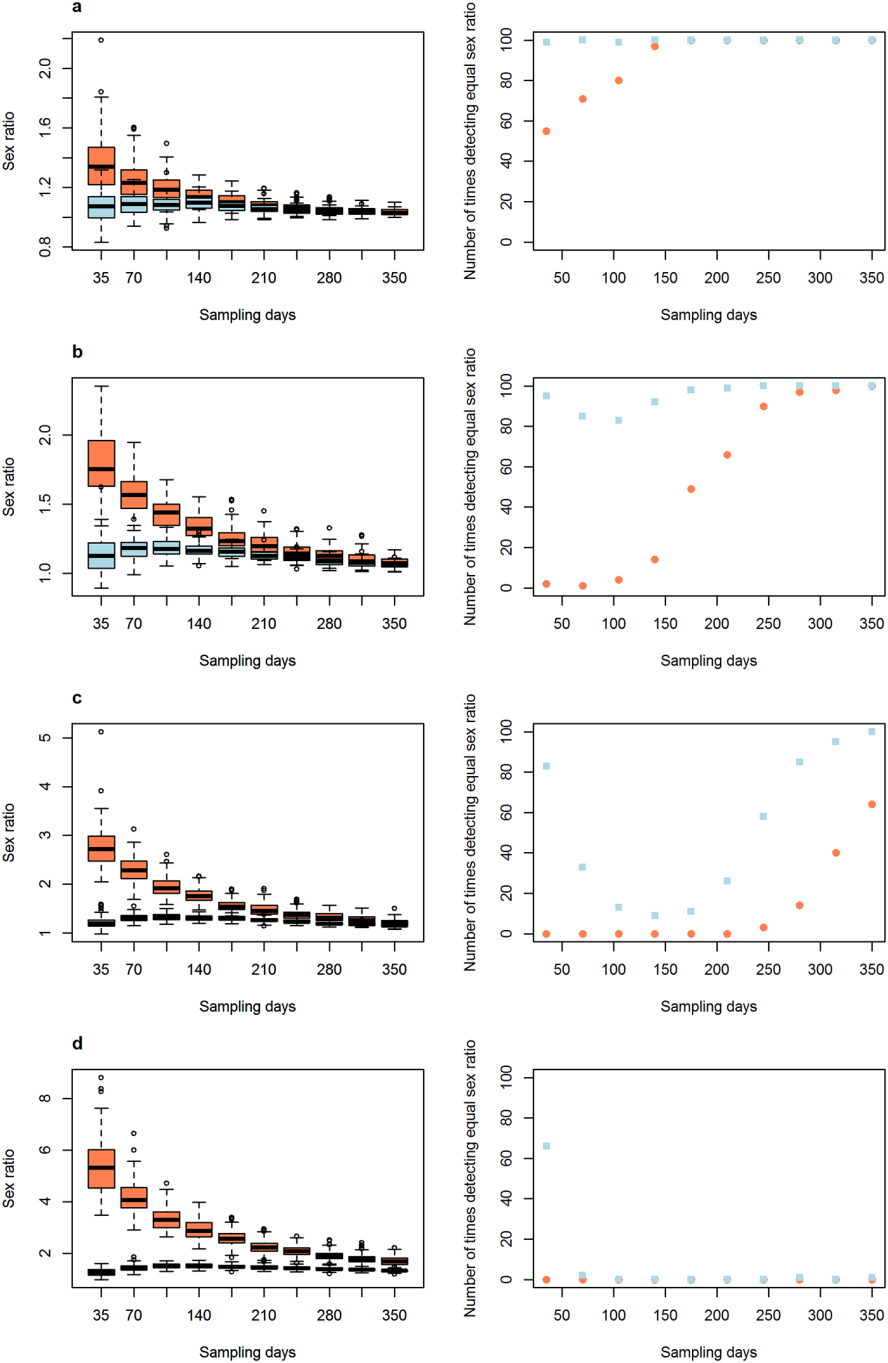
The influence of number of sampling days on the efficiency of the sampling methods in estimating a population’s sex ratio when there are differences in the movement pattern between males and females. Left graphs represent sex ratio distributions and right graphs represent the number of times the sex ratio was recorded as equal in 100 simulations with varying sampling days. From (a) to (d), increasing difference in movement pattern: a) movement probability of males = 0.6 and females = 0.4; b) males = 0.7 and females = 0.3; c) males = 0.8 and females = 0.2; d) males = 0.9 and females = 0.1. The number of times sex ratio was recorded as equal was determined by the number of times that the chi-square test performed on the sex ratio sampled in each simulation found a non-significant result. Red circles represent passive capture methods, and blue squares are active searches.

**Fig 3:**
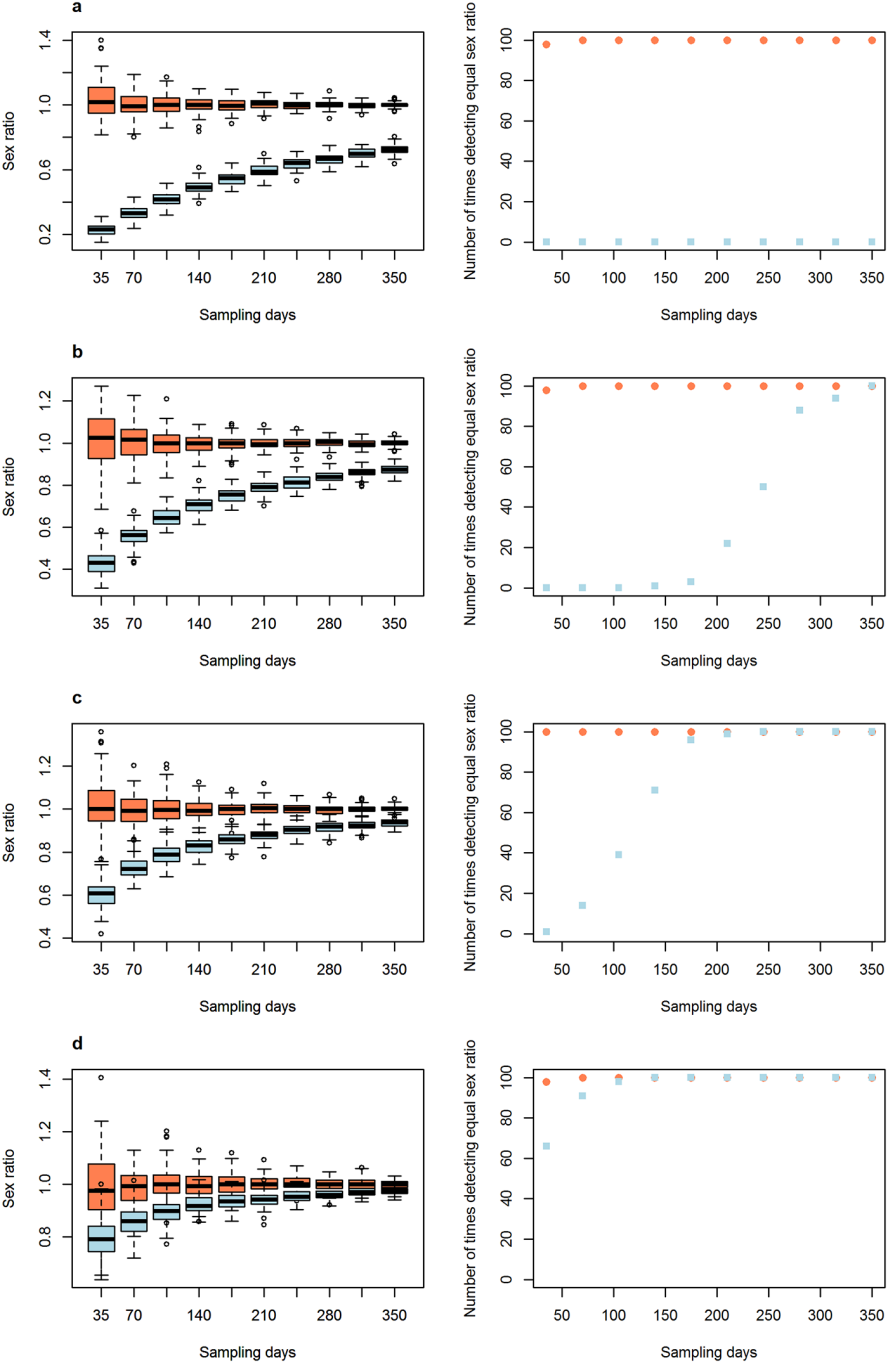
The influence of number of sampling days on the efficiency of the sampling methods in estimating a population’s sex ratio when there are differences in detectability between males and females. Left graphs represent sex ratio distributions and right graphs represent the number of times the sex ratio was recorded as equal in 100 simulations with varying sampling days. From (a) to (d), decreasing difference in detectability: a) males = 0.1 and females = 0.9; b) males = 0.2 and females = 0.8; c) males = 0.3 and females = 0.7; d) males = 0.4 and females = 0.6. The number of times sex ratio was recorded as equal was determined by the number of times that the chi-square test performed on the sex ratio sampled in each simulation found a non-significant result. Red circles represent passive capture methods, and blue squares are active searches. Note the efficiency in pitfall traps is constant during the simulations, since detectability implemented in our simulation is related only to active searches.

**Fig 4:**
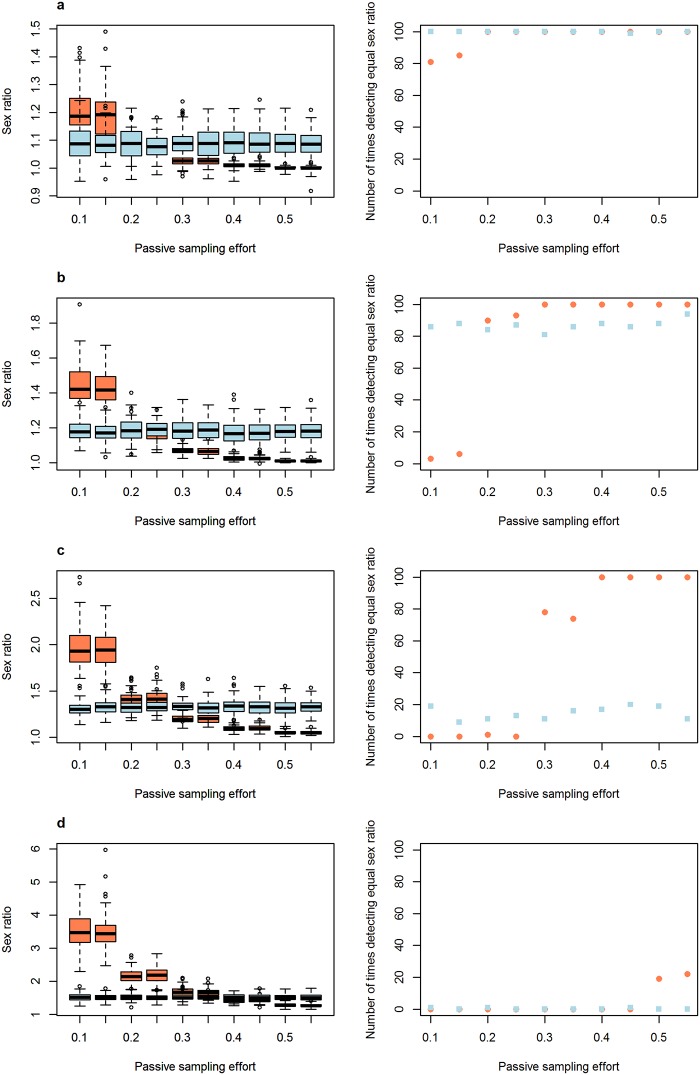
The influence of passive sampling effort on the efficiency of the sampling methods in estimating a population’s sex ratio when there are differences in movement pattern between males and females. Left graphs represent sex ratio distributions and right graphs represent the number of times the sex ratio was recorded as equal in 100 simulations with varying passive sampling efforts. From (a) to (d), increasing difference in movement pattern. a) male movement probability = 0.6 and female = 0.4; b) male = 0.7 and female = 0.3; c) male = 0.8 and female = 0.2; d) male = 0.9 and female = 0.1. The number of times sex ratio was recorded as equal was determined by the number of times that the chi-square test performed on the sex ratio sampled in each simulation found a non-significant result. Red circles represent passive capture methods, and blue squares are active searches.

The second set of parameters that were evaluated to see how sampling days and sampling effort influenced the biases generated by differences in movement pattern and detectability had interesting results. Variations in movement pattern had a stronger effect on passive traps than on active searches (Fig 2), and active searches needed less sampling days to recover the equal sex ratio than passive searches. Variations in detectability obviously had an effect only on active searches (Fig 3). This effect was very strong when one of the sexes was nine times more likely to be detected than the other (Fig 3a), but this effect reduced and eventually disappeared when the number of sampling days increased (Fig 3b–3d). Finally, an increase in passive sampling effort allowed a more accurate evaluation of a population’s sex ratio when movement patterns between the sexes varied (Fig 4). When passive sampling effort was high, passive capture methods were more efficient than active searches in recovering the equal sex ratio.

## Discussion

The efficiency of passive capture methods and active searches to correctly estimate a population’s sex ratio, according to our findings, was highly dependent on passive sampling effort and number of sampling days when there were differences in movement pattern and detectability between males and females. To our knowledge, this is the first study addressing how efficient these commonly used sampling methods in biodiversity studies are in recovering unbiased sex ratios.

Passive capture methods, such as pitfall traps and mist nets, are commonly used in biodiversity studies of vertebrates, and many studies support the efficiency of these methods in correctly sampling the diversity [3,4,7–9]. However, our results highlight that passive methods may not provide good estimates of a population’s sex ratio when sampling effort (number of sampling days and/or number of passive traps) is low. Since the costs related to installing pitfall traps (financial and time) are high [7], keeping these traps active for more time may be the better solution to properly sample the sex ratio of the studied species.

Active searches were more efficient in recovering a population’s sex ratio when considering variations in movement patterns between the sexes than passive methods, but the high-dependency of this method on detectability could be problematic. This is particular common in active searches for anurans and birds, where the survey is commonly guided by vocalisations, which are a pervasive activity of males [17,18]. As a result, a male-biased sex ratio is common [16,17], although biases may also be due to biological characteristics of the species, such as differences in maturation time [16]. Therefore, in such cases when differences in detectability are evident and when the researcher suspects a high disparity in this characteristic, a greater sampling effort in terms of the number of days of active searches should be performed, as shown in Fig 3.

Our results suggest that data from passive sampling and active searches may be used to infer differences in movement pattern between males and females. When detectability is equal for males and females, active searches should result in a good estimate of the population’s sex ratio under intermediate to high sampling effort (number of sampling days), even when differences in movement pattern are as large as four times (Fig 2a–2c). A high sampling effort may also ensure that a population’s sex ratio estimate from active searches is accurate even when differences in detectability are as large as four times (Fig 3b–3d). On the other hand, passive methods may be used to infer differences in movement patterns between the sexes because, when such differences exist, passive methods will mainly recover the difference in movement pattern instead of the sex ratio of the population (Fig 2). Then, if active searches are conducted with medium to high sampling day effort and they find equal sex ratios, but passive sampling finds biased sex ratios, the sex most sampled in passive sampling is the most active. Such approximations are not able to recover the exact difference in movement pattern between males and females, but they provide valuable insights regarding which sex is more active. Hence, this approach has an interesting unexploited value as it allows us to use data from simple capture methods used in most studies to infer general movement patterns, which are commonly inferred from the use of expensive tools, such as radio-trackers. Thus, our simulation offers important guidelines to follow when sampling in the real world.

Our simulation approach allowed us to recognise all captured individuals, avoiding the risk of counting the same individual multiple times, which is similar to a mark-recapture technique. Then, the applicability of our results to field studies is appropriate only in situations where individuals are marked or removed from the population, so they cannot be counted multiple times.

The simulation model here developed has two additional limitations that were not accounted for and could be addressed in future studies: differences in microhabitat use and differences in trappability between males and females. Differences in microhabitat use are common in many animal species [19–21], and these differences could also generate biases in capture results from field studies, mainly for passive methods which use fixed traps. However, considering that the studied area was thoroughly sampled and consequently, the different microhabitats were fully explored, such biases may not have such a strong effect. Differences in trappability, where males or females are more capture-prone or capture shy [22], may also occur and influence the results of passive methods. Future studies could incorporate this parameter in the model and evaluate its effect.

We conclude that active searches and passive capture methods are able to correctly recover the population’s sex ratio, but differences in movement pattern and detectability between males and females may require a high sampling effort to ensure an unbiased estimate. Thus, prior knowledge regarding movement pattern and detectability for the species is important information to guide field studies aiming to understand sex ratio related patterns.

## Supporting Information

S1 Appendix R code used in the simulation performed in our study. (TXT)

S1 Fig Visual representation of passive (a) and active (b) sampling methods in a 8x8 geographical domain. Both sampling methods are established in random transects in the geographical domain. For the passive sampling method (a) random latitude coordinates (y) are chosen to install the sampling stations (y was defined randomly in this figure as equal to 4). The number of transects of the passive capture method depends on the passive sampling effort defined. In this visual representation, the passive sampling effort was 0.10, which represents a number of transects equal to 10% of the grid resolution. In the 8x8 geographical domain the number of transects are rounded to one, explaining why only one y coordinate was chosen. After defining the random y coordinate, an x coordinate is randomly chosen varying from one to half the geographical domain dimension (the randomly defined value of x in this figure was x = 4). Transects have a fixed length of half of the grid resolution, and sampling stations are located in individual cells of the transect, alternating one cell containing a trap with the next without a trap. For the active search (b), the sampling processes for randomly choosing y and x are repeated (y = 6, x = 3). However, the active searches were designed to visit all cells corresponding to the randomly established transect and the fixed number of cells sampled are half of the geographical domain dimension.
(PDF)

S1 Table Parameters used in our simulation to evaluate how grid size (R), number of sampling days or time steps (Days), sampling effort (SaEf), difference in movement pattern between males and females (Mov; Mov = 0.5 indicates that both sexes have the same movement pattern), detectability of males (DeMa) and females (DeFe) influence the number of sampled sex ratios biased towards males (MaPS), females (FePS), and unbiased (UnbiasedPS) in passive sampling methods and biased toward males (MaAS), females (FeAS), and unbiased (UnbiasedAS) in active sampling methods. Population’s sex ratio was always equal in our simulations (same number of males and females). We performed 100 runs for each parameter combination (note that MaPS + FePS + UnbiasedPS = 100 for each parameter combination, the same is valid for active search parameters), first with 100 time steps without sampling in order to allow individuals to move around the grid according to their movement pattern; and then 2) 100 time steps of sampling with both capture methods. We used chi-square tests to evaluate if the sampled sex ratio was significantly different from 1:1 in each run.
(PDF)

S2 Table Parameters used in our simulation to evaluate how number of sampling days or time steps (Days) and sampling effort (SaEf) influence the number of sampled sex ratios biased toward males (MaPS), females (FePS), and unbiased (UnbiasedPS) in passive sampling methods and biased toward males (MaAS), females (FeAS), and unbiased (UnbiasedAS) in active sampling methods, when males and females have different movement patterns (Mov: Mov = 0.5 represents both sexes having the same movement pattern) and detectability (DeMa and DeFe). R is the grid size (number of rows and columns). Population’s sex ratio was always equal in our simulations (same number of males and females). We performed 100 runs for each parameter combination (note that MaPS + FePS + UnbiasedPS = 100 for each parameter combination, the same is valid for active search parameters) with 100 time steps previous to sampling in order to allow individuals to move around the grid according to their movement pattern; and then 2) 100 time steps of sampling with both capture methods. We used chi-square tests to evaluate if the sampled sex ratio was significantly different from 1:1 in each run.
(PDF)
