# Supplementary material for: Differences in Movement Pattern and Detectability between Males and Females Influence How Common Sampling Methods Estimate Sex Ratio
Source: PLoS One. 2016 Jul 21;11(7):e0159736. doi: 10.1371/journal.pone.0159736 (PMC4956211; doi:10.1371/journal.pone.0159736)
Supplement: S1 Fig — Both sampling methods are established in random transects in the geographical domain. For the passive sampling method (a) random latitude coordinates (y) are chosen to install the sampling stations (y was defined randomly in this figure as equal to 4). The number of transects of the passive capture method depends on the passive sampling effort defined. In this visual representation, the passive sampling effort was 0.10, which represents a number of transects equal to 10% of the grid resolution. In the 8x8 geographical domain the number of transects are rounded to one, explaining why only one y coordinate was chosen. After defining the random y coordinate, an x coordinate is randomly chosen varying from one to half the geographical domain dimension (the randomly defined value of x in this figure was x = 4). Transects have a fixed length of half of the grid resolution, and sampling stations are located in individual cells of the transect, alternating one cell containing a trap with the next without a trap. For the active search (b), the sampling processes for randomly choosing y and x are repeated (y = 6, x = 3). However, the active searches were designed to visit all cells corresponding to the randomly established transect and the fixed number of cells sampled are half of the geographical domain dimension. (PDF) [file pone.0159736.s002.pdf]

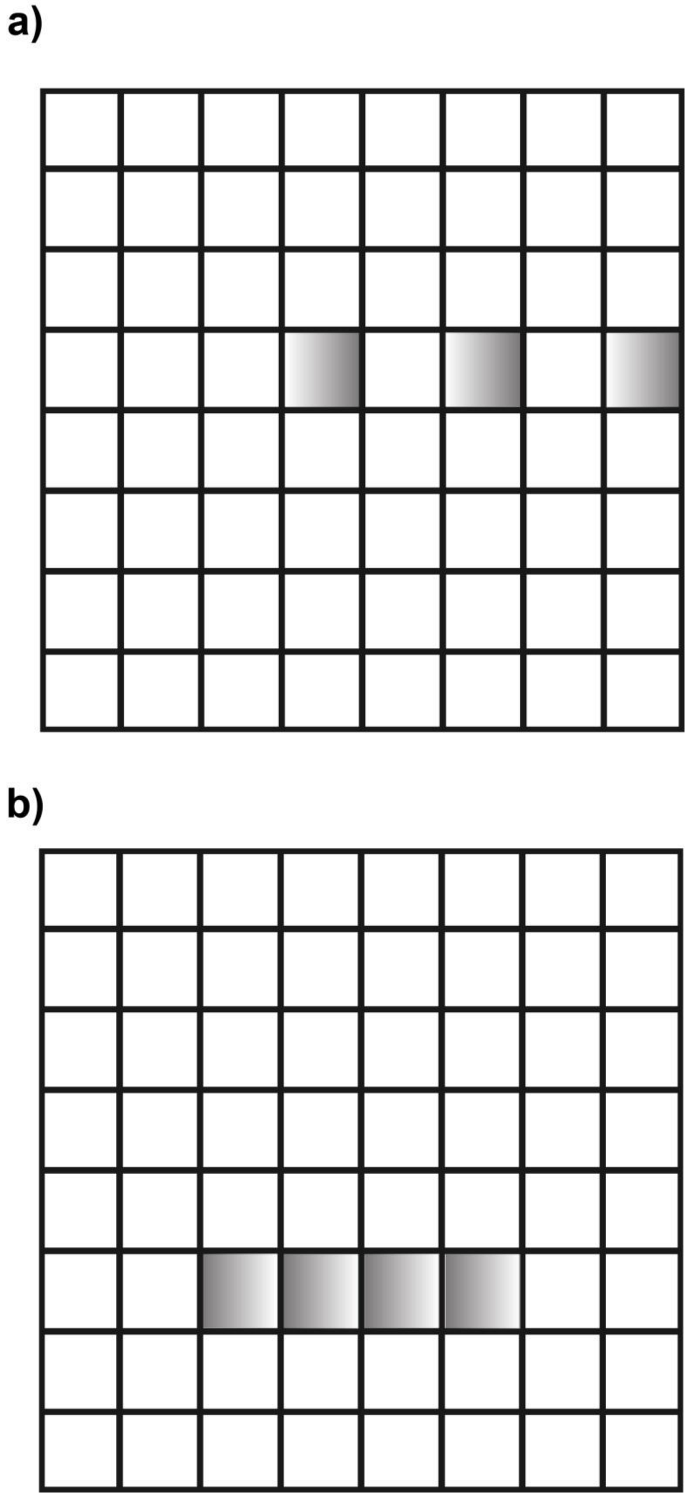

S1 Fig. Visual representation of passive (a) and active (b) sampling methods in a 8x8 geographical domain. Both sampling methods are established in random transects in the geographical domain. For the passive sampling method (a) random latitude coordinates (y) are chosen to install the sampling stations (y was defined randomly in this figure as equal to 4). The number of transects of the passive capture method depends on the passive sampling effort defined. In this visual representation, the passive

sampling effort was 0.10, which represents a number of transects equal to 10% of the grid resolution. In the 8x8 geographical domain the number of transects are rounded to one, explaining why only one y coordinate was chosen. After defining the random y coordinate, an x coordinate is randomly chosen varying from one to half the geographical domain dimension (the randomly defined value of x in this figure was x=4). Transects have a fixed length of half of the grid resolution, and sampling stations are located in individual cells of the transect, alternating one cell containing a trap with the next without a trap. For the active search (b), the sampling processes for randomly choosing y and x are repeated (y = 6, x = 3). However, the active searches were designed to visit all cells corresponding to the randomly established transect and the fixed number of cells sampled are half of the geographical domain dimension.
