# Supplementary material for: Differences in Movement Pattern and Detectability between Males and Females Influence How Common Sampling Methods Estimate Sex Ratio
Source: PLoS One. 2016 Jul 21;11(7):e0159736. doi: 10.1371/journal.pone.0159736 (PMC4956211; doi:10.1371/journal.pone.0159736)
Supplement: S1 Table — Population’s sex ratio was always equal in our simulations (same number of males and females). We performed 100 runs for each parameter combination (note that MaPS + FePS + UnbiasedPS = 100 for each parameter combination, the same is valid for active search parameters), first with 100 time steps without sampling in order to allow individuals to move around the grid according to their movement pattern; and then 2) 100 time steps of sampling with both capture methods. We used chi-square tests to evaluate if the sampled sex ratio was significantly different from 1:1 in each run. (PDF) [file pone.0159736.s003.pdf]

S1 Table. Parameters used in our simulation to evaluate how grid size (R), number of sampling days or time steps (Days), sampling effort (SaEf), difference in movement pattern between males and females (Mov; Mov = 0.5 indicates that both sexes have the same movement pattern), detectability of males (DeMa) and females (DeFe) influence the number of sampled sex ratios biased towards males (MaPS), females (FePS), and unbiased (UnbiasedPS) in passive sampling methods and biased toward males (MaAS), females (FeAS), and unbiased (UnbiasedAS) in active sampling methods. Population's sex ratio was always equal in our simulations (same number of males and females). We performed 100 runs for each parameter combination (note that MaPS + FePS + UnbiasedPS = 100 for each parameter combination, the same is valid for active search parameters), first with 100 time steps without sampling in order to allow individuals to move around the grid according to their movement pattern; and then 2) 100 time steps of sampling with both capture methods. We used chi-square tests to evaluate if the sampled sex ratio was significantly different from 1:1 in each run.

R	Days	Mov	SaEf	DeMa	DeFe	MaPS	FePS	UnbiasedPS	MaAS	FeAS	UnbiasedAS
10	105	0.5	0.1	0.5	0.5	0	0	100	0	0	100
15	105	0.5	0.1	0.5	0.5	0	0	100	0	0	100
20	105	0.5	0.1	0.5	0.5	0	0	100	0	0	100
25	105	0.5	0.1	0.5	0.5	0	0	100	0	0	100
30	105	0.5	0.1	0.5	0.5	0	0	100	1	0	99
35	105	0.5	0.1	0.5	0.5	0	0	100	0	1	99
40	105	0.5	0.1	0.5	0.5	0	0	100	0	2	98
45	105	0.5	0.1	0.5	0.5	0	0	100	0	1	99
50	105	0.5	0.1	0.5	0.5	0	0	100	2	0	98
55	105	0.5	0.1	0.5	0.5	0	0	100	0	1	99
10	35	0.5	0.1	0.5	0.5	0	1	99	0	0	100
10	70	0.5	0.1	0.5	0.5	0	0	100	0	0	100
10	105	0.5	0.1	0.5	0.5	0	0	100	0	0	100

R	Days	Mov	SaEf	DeMa	DeFe	MaPS	FePS	UnbiasedPS	MaAS	FeAS	UnbiasedAS
10	140	0.5	0.1	0.5	0.5	0	0	100	0	0	100
10	175	0.5	0.1	0.5	0.5	0	0	100	0	0	100
10	210	0.5	0.1	0.5	0.5	0	0	100	0	0	100
10	245	0.5	0.1	0.5	0.5	0	0	100	0	0	100
10	280	0.5	0.1	0.5	0.5	0	0	100	0	0	100
10	315	0.5	0.1	0.5	0.5	0	0	100	0	0	100
10	350	0.5	0.1	0.5	0.5	0	0	100	0	0	100
10	105	0.5	0.1	0.5	0.5	0	0	100	0	0	100
10	105	0.55	0.1	0.5	0.5	1	0	99	0	0	100
10	105	0.6	0.1	0.5	0.5	15	0	85	0	0	100
10	105	0.65	0.1	0.5	0.5	66	0	34	8	0	92
10	105	0.7	0.1	0.5	0.5	98	0	2	15	0	85
10	105	0.75	0.1	0.5	0.5	100	0	0	51	0	49
10	105	0.8	0.1	0.5	0.5	100	0	0	85	0	15
10	105	0.85	0.1	0.5	0.5	100	0	0	100	0	0
10	105	0.9	0.1	0.5	0.5	100	0	0	100	0	0
10	105	0.95	0.1	0.5	0.5	100	0	0	100	0	0
10	105	0.5	0.1	0.5	0.5	0	0	100	0	0	100
10	105	0.5	0.15	0.5	0.5	0	0	100	0	0	100
10	105	0.5	0.2	0.5	0.5	0	0	100	0	0	100
10	105	0.5	0.25	0.5	0.5	0	0	100	0	0	100
10	105	0.5	0.3	0.5	0.5	0	0	100	0	0	100
10	105	0.5	0.35	0.5	0.5	0	0	100	0	0	100
10	105	0.5	0.4	0.5	0.5	0	0	100	0	0	100
10	105	0.5	0.45	0.5	0.5	0	0	100	0	0	100
10	105	0.5	0.5	0.5	0.5	0	0	100	0	0	100
10	105	0.5	0.55	0.5	0.5	0	0	100	0	0	100

R	Days	Mov	SaEf	DeMa	DeFe	MaPS	FePS	UnbiasedPS	MaAS	FeAS	UnbiasedAS
10	105	0.5	0.1	0.1	0.1	0	0	100	1	2	97
10	105	0.5	0.1	0.2	0.2	0	0	100	0	0	100
10	105	0.5	0.1	0.3	0.3	0	0	100	0	0	100
10	105	0.5	0.1	0.4	0.4	0	0	100	0	0	100
10	105	0.5	0.1	0.5	0.5	0	0	100	0	0	100
10	105	0.5	0.1	0.6	0.6	0	0	100	0	0	100
10	105	0.5	0.1	0.7	0.7	0	0	100	0	0	100
10	105	0.5	0.1	0.8	0.8	0	0	100	0	0	100
10	105	0.5	0.1	0.9	0.9	0	0	100	0	0	100
10	105	0.5	0.1	1	1	0	0	100	0	0	100
10	105	0.5	0.1	0.1	0.9	0	0	100	0	100	0
10	105	0.5	0.1	0.2	0.8	0	1	99	0	100	0
10	105	0.5	0.1	0.3	0.7	0	0	100	0	52	48
10	105	0.5	0.1	0.4	0.6	0	0	100	0	0	100
10	105	0.5	0.1	0.5	0.5	0	0	100	0	0	100
10	105	0.5	0.1	0.6	0.4	0	0	100	0	0	100
10	105	0.5	0.1	0.7	0.3	0	0	100	60	0	40
10	105	0.5	0.1	0.8	0.2	0	0	100	100	0	0
10	105	0.5	0.1	0.9	0.1	0	0	100	100	0	0
