# Supplementary material for: Differences in Movement Pattern and Detectability between Males and Females Influence How Common Sampling Methods Estimate Sex Ratio
Source: PLoS One. 2016 Jul 21;11(7):e0159736. doi: 10.1371/journal.pone.0159736 (PMC4956211; doi:10.1371/journal.pone.0159736)
Supplement: S2 Table — R is the grid size (number of rows and columns). Population’s sex ratio was always equal in our simulations (same number of males and females). We performed 100 runs for each parameter combination (note that MaPS + FePS + UnbiasedPS = 100 for each parameter combination, the same is valid for active search parameters) with 100 time steps previous to sampling in order to allow individuals to move around the grid according to their movement pattern; and then 2) 100 time steps of sampling with both capture methods. We used chi-square tests to evaluate if the sampled sex ratio was significantly different from 1:1 in each run. (PDF) [file pone.0159736.s004.pdf]

S2 Table. Parameters used in our simulation to evaluate how number of sampling days or time steps (Days) and sampling effort (SaEf) influence the number of sampled sex ratios biased toward males (MaPS), females (FePS), and unbiased (UnbiasedPS) in passive sampling methods and biased toward males (MaAS), females (FeAS), and unbiased (UnbiasedAS) in active sampling methods, when males and females have different movement patterns (Mov: Mov = 0.5 represents both sexes having the same movement pattern) and detectability (DeMa and DeFe). R is the grid size (number of rows and columns). Population's sex ratio was always equal in our simulations (same number of males and females). We performed 100 runs for each parameter combination (note that MaPS + FePS + UnbiasedPS = 100 for each parameter combination, the same is valid for active search parameters) with 100 time steps previous to sampling in order to allow individuals to move around the grid according to their movement pattern; and then 2) 100 time steps of sampling with both capture methods. We used chi-square tests to evaluate if the sampled sex ratio was significantly different from 1:1 in each run.

R	Days	Mov	SaEf	DeMa	DeFe	MaPS	FePS	UnbiasedPS	MaAS	FeAS	UnbiasedAS
10	35	0.6	0.1	0.5	0.5	45	0	55	1	0	99
10	70	0.6	0.1	0.5	0.5	29	0	71	0	0	100
10	105	0.6	0.1	0.5	0.5	20	0	80	1	0	99
10	140	0.6	0.1	0.5	0.5	3	0	97	0	0	100
10	175	0.6	0.1	0.5	0.5	0	0	100	0	0	100
10	210	0.6	0.1	0.5	0.5	0	0	100	0	0	100
10	245	0.6	0.1	0.5	0.5	0	0	100	0	0	100
10	280	0.6	0.1	0.5	0.5	0	0	100	0	0	100
10	315	0.6	0.1	0.5	0.5	0	0	100	0	0	100
10	350	0.6	0.1	0.5	0.5	0	0	100	0	0	100
10	35	0.7	0.1	0.5	0.5	98	0	2	5	0	95
10	70	0.7	0.1	0.5	0.5	99	0	1	15	0	85

R	Days	Mov	SaEf	DeMa	DeFe	MaPS	FePS	UnbiasedPS	MaAS	FeAS	UnbiasedAS
10	105	0.7	0.1	0.5	0.5	96	0	4	17	0	83
10	140	0.7	0.1	0.5	0.5	86	0	14	8	0	92
10	175	0.7	0.1	0.5	0.5	51	0	49	2	0	98
10	210	0.7	0.1	0.5	0.5	34	0	66	1	0	99
10	245	0.7	0.1	0.5	0.5	10	0	90	0	0	100
10	280	0.7	0.1	0.5	0.5	3	0	97	0	0	100
10	315	0.7	0.1	0.5	0.5	2	0	98	0	0	100
10	350	0.7	0.1	0.5	0.5	0	0	100	0	0	100
10	35	0.8	0.1	0.5	0.5	100	0	0	17	0	83
10	70	0.8	0.1	0.5	0.5	100	0	0	67	0	33
10	105	0.8	0.1	0.5	0.5	100	0	0	87	0	13
10	140	0.8	0.1	0.5	0.5	100	0	0	91	0	9
10	175	0.8	0.1	0.5	0.5	100	0	0	89	0	11
10	210	0.8	0.1	0.5	0.5	100	0	0	74	0	26
10	245	0.8	0.1	0.5	0.5	97	0	3	42	0	58
10	280	0.8	0.1	0.5	0.5	86	0	14	15	0	85
10	315	0.8	0.1	0.5	0.5	60	0	40	5	0	95
10	350	0.8	0.1	0.5	0.5	36	0	64	0	0	100
10	35	0.9	0.1	0.5	0.5	100	0	0	34	0	66
10	70	0.9	0.1	0.5	0.5	100	0	0	98	0	2
10	105	0.9	0.1	0.5	0.5	100	0	0	100	0	0
10	140	0.9	0.1	0.5	0.5	100	0	0	100	0	0
10	175	0.9	0.1	0.5	0.5	100	0	0	100	0	0
10	210	0.9	0.1	0.5	0.5	100	0	0	100	0	0
10	245	0.9	0.1	0.5	0.5	100	0	0	100	0	0
10	280	0.9	0.1	0.5	0.5	100	0	0	99	0	1
10	315	0.9	0.1	0.5	0.5	100	0	0	100	0	0

R	Days	Mov	SaEf	DeMa	DeFe	MaPS	FePS	UnbiasedPS	MaAS	FeAS	UnbiasedAS
10	350	0.9	0.1	0.5	0.5	100	0	0	99	0	1
10	35	0.5	0.1	0.1	0.9	2	0	98	0	100	0
10	70	0.5	0.1	0.1	0.9	0	0	100	0	100	0
10	105	0.5	0.1	0.1	0.9	0	0	100	0	100	0
10	140	0.5	0.1	0.1	0.9	0	0	100	0	100	0
10	175	0.5	0.1	0.1	0.9	0	0	100	0	100	0
10	210	0.5	0.1	0.1	0.9	0	0	100	0	100	0
10	245	0.5	0.1	0.1	0.9	0	0	100	0	100	0
10	280	0.5	0.1	0.1	0.9	0	0	100	0	100	0
10	315	0.5	0.1	0.1	0.9	0	0	100	0	100	0
10	350	0.5	0.1	0.1	0.9	0	0	100	0	100	0
10	35	0.5	0.1	0.2	0.8	0	2	98	0	100	0
10	70	0.5	0.1	0.2	0.8	0	0	100	0	100	0
10	105	0.5	0.1	0.2	0.8	0	0	100	0	100	0
10	140	0.5	0.1	0.2	0.8	0	0	100	0	99	1
10	175	0.5	0.1	0.2	0.8	0	0	100	0	97	3
10	210	0.5	0.1	0.2	0.8	0	0	100	0	78	22
10	245	0.5	0.1	0.2	0.8	0	0	100	0	50	50
10	280	0.5	0.1	0.2	0.8	0	0	100	0	12	88
10	315	0.5	0.1	0.2	0.8	0	0	100	0	6	94
10	350	0.5	0.1	0.2	0.8	0	0	100	0	0	100
10	35	0.5	0.1	0.3	0.7	0	0	100	0	99	1
10	70	0.5	0.1	0.3	0.7	0	0	100	0	86	14
10	105	0.5	0.1	0.3	0.7	0	0	100	0	61	39
10	140	0.5	0.1	0.3	0.7	0	0	100	0	29	71
10	175	0.5	0.1	0.3	0.7	0	0	100	0	4	96
10	210	0.5	0.1	0.3	0.7	0	0	100	0	1	99

R	Days	Mov	SaEf	DeMa	DeFe	MaPS	FePS	UnbiasedPS	MaAS	FeAS	UnbiasedAS
10	245	0.5	0.1	0.3	0.7	0	0	100	0	0	100
10	280	0.5	0.1	0.3	0.7	0	0	100	0	0	100
10	315	0.5	0.1	0.3	0.7	0	0	100	0	0	100
10	350	0.5	0.1	0.3	0.7	0	0	100	0	0	100
10	35	0.5	0.1	0.4	0.6	1	1	98	0	34	66
10	70	0.5	0.1	0.4	0.6	0	0	100	0	9	91
10	105	0.5	0.1	0.4	0.6	0	0	100	0	2	98
10	140	0.5	0.1	0.4	0.6	0	0	100	0	0	100
10	175	0.5	0.1	0.4	0.6	0	0	100	0	0	100
10	210	0.5	0.1	0.4	0.6	0	0	100	0	0	100
10	245	0.5	0.1	0.4	0.6	0	0	100	0	0	100
10	280	0.5	0.1	0.4	0.6	0	0	100	0	0	100
10	315	0.5	0.1	0.4	0.6	0	0	100	0	0	100
10	350	0.5	0.1	0.4	0.6	0	0	100	0	0	100
10	105	0.6	0.1	0.5	0.5	19	0	81	0	0	100
10	105	0.6	0.15	0.5	0.5	15	0	85	0	0	100
10	105	0.6	0.2	0.5	0.5	0	0	100	0	0	100
10	105	0.6	0.25	0.5	0.5	0	0	100	0	0	100
10	105	0.6	0.3	0.5	0.5	0	0	100	0	0	100
10	105	0.6	0.35	0.5	0.5	0	0	100	0	0	100
10	105	0.6	0.4	0.5	0.5	0	0	100	0	0	100
10	105	0.6	0.45	0.5	0.5	0	0	100	1	0	99
10	105	0.6	0.5	0.5	0.5	0	0	100	0	0	100
10	105	0.6	0.55	0.5	0.5	0	0	100	0	0	100
10	105	0.7	0.1	0.5	0.5	97	0	3	14	0	86
10	105	0.7	0.15	0.5	0.5	94	0	6	12	0	88
10	105	0.7	0.2	0.5	0.5	10	0	90	16	0	84

R	Days	Mov	SaEf	DeMa	DeFe	MaPS	FePS	UnbiasedPS	MaAS	FeAS	UnbiasedAS
10	105	0.7	0.25	0.5	0.5	7	0	93	13	0	87
10	105	0.7	0.3	0.5	0.5	0	0	100	19	0	81
10	105	0.7	0.35	0.5	0.5	0	0	100	14	0	86
10	105	0.7	0.4	0.5	0.5	0	0	100	12	0	88
10	105	0.7	0.45	0.5	0.5	0	0	100	14	0	86
10	105	0.7	0.5	0.5	0.5	0	0	100	12	0	88
10	105	0.7	0.55	0.5	0.5	0	0	100	6	0	94
10	105	0.8	0.1	0.5	0.5	100	0	0	81	0	19
10	105	0.8	0.15	0.5	0.5	100	0	0	91	0	9
10	105	0.8	0.2	0.5	0.5	99	0	1	89	0	11
10	105	0.8	0.25	0.5	0.5	100	0	0	87	0	13
10	105	0.8	0.3	0.5	0.5	22	0	78	89	0	11
10	105	0.8	0.35	0.5	0.5	26	0	74	84	0	16
10	105	0.8	0.4	0.5	0.5	0	0	100	83	0	17
10	105	0.8	0.45	0.5	0.5	0	0	100	80	0	20
10	105	0.8	0.5	0.5	0.5	0	0	100	81	0	19
10	105	0.8	0.55	0.5	0.5	0	0	100	89	0	11
10	105	0.9	0.1	0.5	0.5	100	0	0	99	0	1
10	105	0.9	0.15	0.5	0.5	100	0	0	100	0	0
10	105	0.9	0.2	0.5	0.5	100	0	0	99	0	1
10	105	0.9	0.25	0.5	0.5	100	0	0	100	0	0
10	105	0.9	0.3	0.5	0.5	100	0	0	100	0	0
10	105	0.9	0.35	0.5	0.5	100	0	0	100	0	0
10	105	0.9	0.4	0.5	0.5	100	0	0	100	0	0
10	105	0.9	0.45	0.5	0.5	100	0	0	99	0	1
10	105	0.9	0.5	0.5	0.5	81	0	19	100	0	0
10	105	0.9	0.55	0.5	0.5	78	0	22	100	0	0
